# A novel calcium fluorinated alkoxyaluminate salt as a next step towards Ca metal anode rechargeable batteries†

**DOI:** 10.1039/d3ta02084c

**Published:** 2023-05-23

**Authors:** Tjaša Pavčnik, Juan D. Forero-Saboya, Alexandre Ponrouch, Ana Robba, Robert Dominko, Jan Bitenc

**Affiliations:** a National Institute of Chemistry Hajdrihova 19 1000 Ljubljana Slovenia jan.bitenc@ki.si; b Faculty of Chemistry and Chemical Technology, University of Ljubljana Večna Pot 113 1000 Ljubljana Slovenia; c Institut de Ciència de Materials de Barcelona, ICMAB-CSIC, Campus UAB Bellaterra 08193 Spain; d Alistore-European Research Institute, CNRS FR 3104, Hub de L'énergie Rue Baudelocque Amiens 80039 France

## Abstract

Ca metal anode rechargeable batteries are seen as a sustainable high-energy density and high-voltage alternative to the current Li-ion battery technology due to the low redox potential of Ca metal and abundance of Ca. Electrolytes are key enablers on the path towards next-generation battery systems. Within this work, we synthesize a new calcium tetrakis(hexafluoroisopropyloxy) aluminate salt, Ca[Al(hfip)_4_]_2_, and benchmark it *versus* the state-of-the-art boron analogue Ca[B(hfip)_4_]_2_. The newly developed aluminate-based electrolyte exhibits improved performance in terms of conductivity, Ca plating/stripping efficiency, and oxidative stability as well as Ca battery cell performance. A marked improvement of 0.5 V higher oxidative stability can pave the path towards high-voltage Ca batteries. A critical issue of solvent quality during salt synthesis is identified as well as solvent decomposition at the Ca metal/electrolyte interface, which leads to passivation of the Ca metal anode. However, the new aluminate salt with preferable electrochemical properties over the existing boron analogue opens up a new area for future Ca battery research based on aluminium compounds.

## Abstract 2

Ca metal anode rechargeable batteries are a good alternative to Li-ion batteries due to abundant Ca metal and its low redox potential. Electrolytes are key for next-gen batteries. We prepared a new Ca[Al(hfip)_4_]_2_ salt and compared it with the state-of-the-art boron analogue.

## Graphical abstract

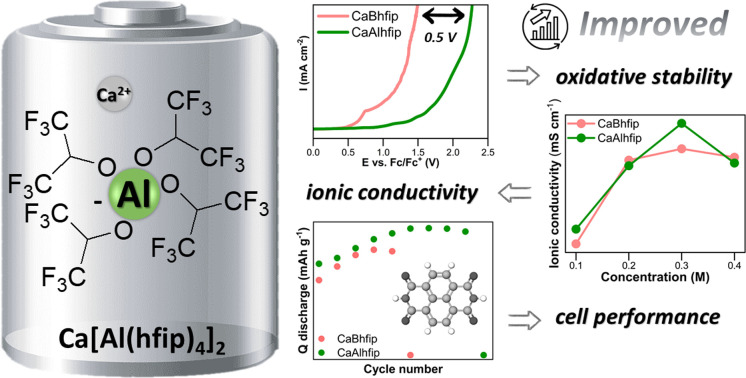

## Introduction

In the last few decades, battery demand has drastically increased, as electronic devices and gadgets have become an indispensable part of our everyday life. Particularly in the last few years, the demand has multiplied due to the electrification of the transport sector and rising stationary storage demand, putting huge pressure on the supply of battery raw materials. Limited abundance of Li as well as several other raw materials (Co, Ni, Si, graphite) used in the production of Li-ion batteries,^1,2^ results in more abundant alkali (Na, K) and alkali earth metals (Mg, Ca) being considered as alternative anode materials.^3^ The divalent nature of Mg and Ca metals enables the storage of two electrons per atom, greatly enhancing both their gravimetric and, especially volumetric capacities. Additionally, the low redox potential of the Ca metal anode also makes it one of the most promising alternatives to Li metal and graphite anodes and a highly sought anode material. However, one of the greatest challenges in multivalent systems is the development of electrolytes enabling plating and stripping with high Coulombic efficiency and low overpotential. While significant progress in Mg electrolytes has been made in the last few years, only a handful of Ca electrolytes enabling reversible Ca plating and stripping have been reported in the last few years. This can be attributed to the lower redox potential of Ca metal (−2.87 V *vs.* SHE and −2.38 V for Mg metal) leading to stronger inclination towards passivation and difficult transport of bivalent cations across the passive layer due to insufficient reductive stability of Ca electrolytes.^4,5^

The first systematic studies on Ca electrolytes were reported in 1980, when Staniewicz *et al.* investigated plating and stripping of Ca metal in the Ca(AlCl_4_)_2_–SOCl_2_ electrolyte.^6^ They confirmed Ca stripping, however, due to the almost immediate anode corrosion and formation of the CaCl_2_ passivation layer, no plating could be confirmed. Research was taken up by Meitav and Peled *et al.* who observed Ca plating on the stainless steel electrode with very poor Coulombic efficiency (<10%).^7^ CaCl_2_ passive layer formation was again recognized as the main issue, which prevents reversible stripping/plating. Although it can conduct anions, it completely blocks Ca^2+^ cation transport.^8^ Almost a decade later, Aurbach *et al.* studied the electrochemistry of the Ca metal anode in various electrolytes comprising simple salts (Ca(ClO_4_)_2_, Ca(BF_4_)_2_, TBA(ClO_4_) and TBA(BF_4_) (TBA-tetrabutylammonium)) in different non-protic solvents (acetonitrile, γ-butyrolactone, propylene carbonate and tetrahydrofuran).^9^ The main conclusion of the research was that the formation of a non-conductive passivation layer prevented Ca metal plating in all tested electrolytes, which highlighted the challenge impeding Ca electrolyte development. In 2016, significant progress was made by the demonstration of reversible Ca plating/stripping from Ca(BF_4_)_2_ in EC/PC (EC = ethylene carbonate, PC = propylene carbonate, w/w = 1/1) at elevated temperatures.^10^ The phenomenon was explained by a favorable SEI composition, which conducted Ca^2+^ ions. The SEI mainly contained CaF_2_, but also OH, C=O, C–O and carbonate fragments, and the composition of the SEI did not change after plating/stripping of Ca metal. Afterwards, it was demonstrated that borate (BO_3_) species formed during electrolyte decomposition are actually the key component to facilitate Ca^2+^ transport through the SEI in the Ca(BF_4_)_2_-based electrolyte and also enabled Ca metal plating/stripping in the Ca(TFSI)_2_ based electrolyte.^11^ Even before the elucidation of the role of the borate SEI layer other boron-containing electrolytes were reported as well. The Ca(BH_4_)_2_/THF electrolyte delivered an important breakthrough as the first Ca electrolyte operating at room temperature.^12^ The Ca(BH_4_)_2_-based electrolyte performed with high Coulombic efficiencies (94–96%) on the Au metal electrode, however, BH_4_^−^ as a strong reducing agent significantly limited the electrolyte's oxidative stability to around 2 V *vs.* Ca/Ca^2+^. The passivation layer that forms on the Ca metal anode and protects Ca metal from side reactions was characterized as CaH_2_. Electrochemical performance of Ca(BH_4_)_2_/THF was additionally improved by the LiBH_4_ additive that influences the Ca^2+^ cation coordination shell structure, which lowers electrolyte solvation energy.^13^ Shortly after the introduction and success of Mg electrolytes based on weakly coordinating anions (WCA), specifically Mg[B(hfip)_4_]_2_,^14^ two independent research groups reported a Ca analogue, Ca[B(hfip)_4_]_2_ (hereinafter denoted as CaBhfip).^15,16^ The CaBhfip electrolyte in 1,2-dimethoxyethane (DME) solvent offered a moderate Coulombic efficiency and good oxidative stability. Its non-nucleophilic character enabled application of electrophilic cathodes, such as sulfur^17,18^ and organic materials,^19^ which displayed better electrochemical reversibility compared with previously investigated inorganic cathode materials. From literature reports a conclusion could be reached that reversible Ca plating and stripping can be achieved only in boron-based electrolytes. Due to the chemical similarity between B and Al, alkoxyaluminate-based electrolytes for Ca batteries have been investigated by computational studies and were recognized as potential candidates.^20,21^ Recently, Leon *et al.* reported calcium tetrakis(perfluoro-*tert*-butoxy) aluminate salt, which has displayed inferior Ca plating/stripping performance to the state-of-the-art CaBhfip electrolyte.^22^ Nonetheless, a higher thermodynamic stability of the [Al(hfip)_4_]^−^ anion, as well as superior performance of Mg[Al(hfip)_4_]_2_ salt over its boron analogue motivated us to develop a synthesis procedure for the preparation of Ca[Al(hfip)_4_]_2_ salt (hereinafter denoted as CaAlhfip).^23,24^ In the present work, we introduce a novel CaAlhfip-based electrolyte. The synthesized salt is characterized by IR and NMR spectroscopy. Afterwards, we study electrolyte's physicochemical properties and electrochemical characteristics and compare them with the already established boron analogue, CaBhfip. The morphology and composition of Ca deposits from both electrolytes are studied with SEM and EDX analysis. Finally, we compare the performance of both electrolytes in a Ca metal anode–organic cathode cell setup, using the naphthalene-hydrazine diimide polymer as a cathode material, and suggest future directions of the Ca electrolyte development exploiting improved properties of the aluminate class of electrolytes.

## Experimental

All synthesis procedures, electrolyte preparation, and cell assembly were carried out in an Ar-filled glovebox, with water and oxygen levels below 0.1 ppm.

### Salt synthesis and characterization

Ca(OCH_3_)_2_, Ca(BH_4_)_2_·2THF, and 2 M Al(CH_3_)_3_/toluene were purchased from Sigma-Aldrich and used as received. 1,1,1,3,3,3-Hexafluoropropan-2-ol (HFIP) (Apollo Scientific, 99.9%) and hexane (Carlo Erba) were both dried with 4 Å molecular sieves for 7 days prior to use. DME (Sigma-Aldrich, HPLC grade, 99.9%) underwent an extensive drying procedure that includes drying with 4 Å molecular sieves for 5 days, reflux with Na/K alloy overnight, and fractional distillation. Acetonitrile (ACN, Acros Organics) was dried in a similar way, but without the reflux step due to the incompatibility of the solvent with the Na/K alloy. The final water content of dried solvents determined with Karl Fischer titration (C20 Mettler Toledo) is below 1 ppm for DME and 5 ppm for ACN.

#### Ca[B(hfip)_4_]_2_ salt

Ca[B(hfip)_4_]_2_ salt was synthesized following the literature procedure,^19^ with the modification in the product isolation step. Briefly, Ca(BH_4_)_2_·2THF (2.5 mmol) was dissolved in 5 mL of DME. HFIP (8 eq., 20 mmol) was dropwise added into the stirred solution. After 6 h of stirring under reflux, the solution was concentrated under reduced pressure and gradually added in hexane to precipitate solid. The solid was filtered, and dried under vacuum at 50 °C for 2 days.

#### Ca[Al(hfip)_4_]_2_ salt

Ca(OCH_3_)_2_ (5 mmol) was added to 15 mL of DME. To the obtained dispersion, 2.5 eq. of HFIP was dropwise added. The mixture was refluxed for 3 days and filtered through a PTFE membrane to remove unreacted Ca(OCH_3_)_2_. The clear filtrate was evaporated under the reduced pressure, to obtain solid Ca(hfip)_2_. The solid Ca(hfip)_2_ was dissolved in 10 mL of DME, followed by dropwise addition of Al(CH_3_)_3_/toluene solution (2.2 eq. *vs.* Ca, 11 mmol) and HFIP (3.1 eq. *vs.* Al, 34.1 mmol). The solution was vigorously stirred for 24 hours at room temperature. Proceeding with the equivalent isolation as in the case of CaBhfip, the reaction mixture was concentrated under the reduced pressure and gradually added into hexane. The precipitated salt was filtered and additionally dried under vacuum at 50 °C for 2 days.

IR characterization was performed under an inert atmosphere using an ATR-IR Alpha II (Bruker) equipped with a Ge crystal. All spectra were recorded at room temperature. Measurements were collected and averaged over 48 scans in the range between 3000 and 600 cm^−1^. ^1^H and ^19^F NMR spectra were measured on a Bruker AVANCE NEO 600 MHz NMR spectrometer using DMSO-d_6_ solvent. Chemical shifts are reported in ppm using the residual solvent peak (in ^1^H spectrum) and trifluoroacetic acid (in ^19^F spectrum) as the reference.

### Electrolyte preparation

All electrolytes were prepared by weighing the appropriate amount of CaBhfip or CaAlhfip salt in a measuring flask and diluting them with dry DME up to the mark to obtain 0.1, 0.2, 0.3 and 0.4 M solutions.

### Physicochemical properties

The ionic conductivity of the electrolytes was measured in the FRA-based Multiplexed Conductivity Meter MCM 10 (BioLogic Science Instruments) from 5 to 60 °C (5 °C incremental steps with at least 30 min equilibration time). Viscosity and density were measured in the same temperature range using a Lovis 2000 M/ME and a DMA 4500 from Anton Parr. The cell temperature was regulated within ±0.02 °C. Ultrapure water was used to calibrate the viscometer and densitometer. The uncertainties of the density and viscosity measurements were less than 5 × 10^−5^ g cm^−3^ and 0.5%, respectively.

### Material and cathode preparation

The naphthalene-hydrazine diimide polymer was synthesized following the literature procedure,^25^ with an addition of 5 wt% of multiwalled carbon nanotubes (MWCNTs, NTL C-grade). MWCNTs were dispersed in *p*-chlorophenol at 50 °C using an ultrasound tip for 3 h before addition of other reagents. Cathodes were prepared by mixing the polymer active material with Printex XE2 carbon black and PTFE binder in a 60 : 30 : 10 weight ratio. All the components were added into a ball mill jar with isopropanol and homogenized for 30 minutes on a Retsch PM100 at 300 rpm. The prepared composite was rolled in between a glass plate and a sheet of baking paper to give self-standing electrodes. 12 mm sized electrodes were cut, dried, and transferred into an Ar-filled glovebox. Loading of active materials was 2 mg cm^−2^.

### Electrochemical characterization

Electrochemical testing was performed under galvanostatic mode with a VMP3 potentiostat from Bio-Logic S. A. in 2- and 3-electrode Swagelok type cells. Cells were assembled with three glassy fiber separators (GF/A, Whatman, 260 μm), wetted with approximately 100 (2-electrode cell) or 200 μL (3-electrode cell) of Ca electrolyte. Ca shots (99.5% Alfa Aesar) were shaped into 12 mm round discs, scratched with a spatula and used as a counter electrode (CE). In 3-electrode cells, Ag wire was used as the reference electrode. To calibrate the potential of the reference electrode, ferrocene (10 mM) was introduced into the electrolyte as an internal standard. Cyclic voltammetry tests were performed on a stainless steel (SS) working electrode (WE), with a 25 mV s^−1^ rate. Ca plating was limited by −8.85 mA cm^−2^ current density and Ca stripping with an upper potential limit of 2.5 V *vs.* Ca/Ca^2+^. Coulombic efficiencies for Ca plating/stripping were calculated manually from CV curves by dividing the area under the curve for Ca stripping with the area above the curve for Ca plating. Linear sweep voltammetry (LSV) was carried out in ACN solvent, with scanning potentials from OCV to 3 V *vs.* the Ag reference electrode at 0.1 mV s^−1^. Electrochemical testing of Ca cells with naphthalene-hydrazine diimide polymer-based cathodes was done in galvanostatic mode at 50 mA g^−1^ in a range from 1.5 to 3.5 V.

### SEM/EDX analysis of Ca deposits

Scanning electron microscopy (SEM) and Energy-Dispersive X-ray Spectroscopy (EDX) were performed using a SEM Supra 35 VP from Carl Zeiss at 20 kV with an Ultim Max 100 (Oxford, UK) EDX detector. To prepare samples of Ca deposits, cells with carbon-coated Al foil as the working electrode and Ca metal as the counter electrode were assembled. One of three glassy fibers was exchanged for the Celgard 2400 separator, putting it on the working electrode side to prevent entanglement of separator fibers with Ca metal deposits. Separators were wetted with approximately 100 μL of CaAlhfip/DME or CaBhfip/DME electrolytes. After discharge with 1 mA cm^−2^ current density for 1 h, the cells were transferred back to the glovebox and disassembled. The working electrode with Ca metal deposits was carefully removed from the cell and washed with 2 mL of DME. The samples were transferred to the SEM chamber using a specially designed sample holder in a vacuum to prevent their decomposition in the ambient environment.

## Results

**Fig. 1:**
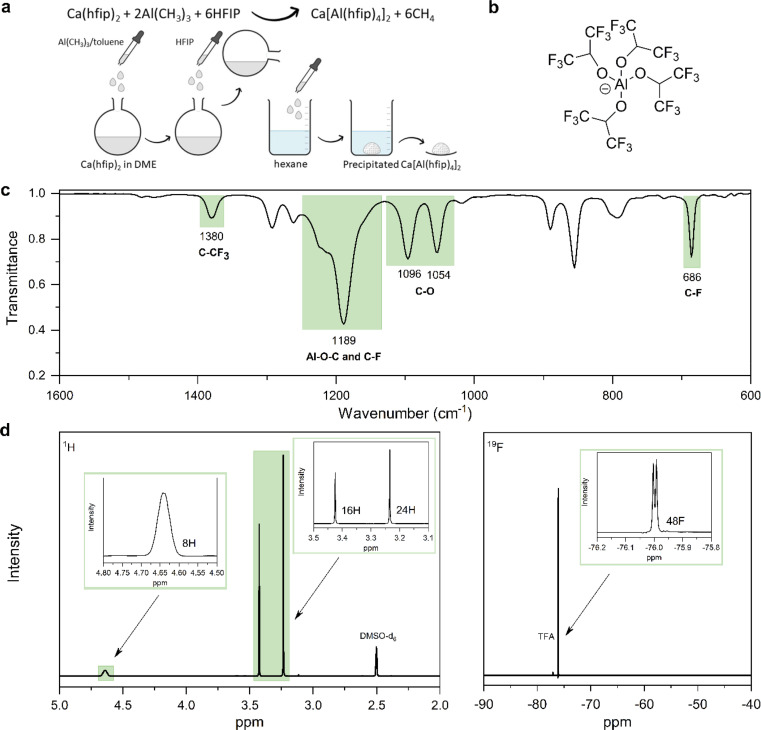
(a) Reaction scheme of CaAlhfip synthesis. (b) Structure of the [Al(hfip)_4_]^−^ anion. (c) ATR-IR spectrum of CaAlhfip with peak assignation. (d) ^1^H (right) and ^19^F (left) NMR spectra of CaAlhfip with peak assignation and integral areas.

Synthesis of Mg alkoxyaluminates from organometallic reagents turned out as an effective way to produce electrolytes with fewer impurities such as H_2_O and O_2_. Organometallic reagents are highly reactive and act as impurity scavengers during salt synthesis.^24^ This motivated the development of the CaAlhfip synthesis procedure where highly reactive Al(CH_3_)_3_ is used as a source of Al. Specifically, CaAlhfip was formed *in situ* from the synthesized Ca(hfip)_2_ by addition of the Al(CH_3_)_3_ and HFIP. The solid product was isolated from the reaction mixture with the salt precipitation in hexane (Fig. 1a). Note that only solvents with high purity should be used for the synthesis and electrolyte preparation. In our work, a significant decrease in electrolyte performance was observed, mainly a decrease of current density during plating and the absence of metal stripping when switching from the chromatographic grade of DME solvent to the synthesis/reagent grade. To better understand this, GC-MS analysis was performed and revealed the presence of high amounts of impurities (Fig. S1†) that were not effectively removed during the drying/purification procedure and were identified as the probable issue limiting the electrolyte performance. Therefore, all the reported experiments in this work refer to the use of the chromatographic grade DME solvent, which, after the drying procedure, offers the highest purity (Fig. S2†).

The obtained CaAlhfip was characterized with ATR-IR and NMR spectroscopy. The IR spectrum (Fig. 1c) shows characteristic peaks of the Alhfip^−^ anion (Fig. 1b).^24^ Al–O–C vibrations, and symmetric and asymmetric stretching of –CF_3_ groups are observed as a broad peak at 1189 cm^−1^, while C–CF_3_ vibrations and –CF_3_ deformations are detected at 1380 and 686 cm^−1^, respectively. C–O stretching vibrations at 1096 and 1054 cm^−1^ originate from both anion and DME solvent molecules that are coordinated on the Ca^2+^ cation. To evaluate the CaAlhfip purity, ^1^H and ^19^F NMR spectra were measured (Fig. 1d). The lack of signals for reactants used in the synthesis confirms their successful conversion. The main signal in the ^1^H spectrum at 4.64 ppm integrates for 8 protons from hexafluoroisopropyloxy groups (–C(H)(CF_3_)_2_) in Alhfip^−^ anions. Additionally, two singlets at 3.24 and 3.42 ppm are observed and attributed –CH_2_– and –CH_3_ groups of DME solvent. Signals integrate for 16 and 24 protons, respectively, which indicate 4 solvent molecules within the salt complex, similar to what was found to be the case with CaBhfip salt.^16^ In the ^19^F spectrum a signal at −76.1 ppm integrates for 48 fluorine atoms originating from two Alhfip^−^ anions. No side products could be detected in NMR. According to the spectroscopy and literature structure of the boron analogue we anticipate the [Ca(DME)_4_][Al(hfip)_4_]_2_ salt structure. To evaluate the performance of novel CaAlhfip salt and compare it with the current state-of-the-art Ca electrolyte, we decided to benchmark it *versus* the boron-based analogue. CaBhfip was synthesized through the previously published procedure, with the additional step of salt precipitation, to improve the purity of the final product and achieve a similar quality of synthesized Al and B salt analogues. Details on the CaBhfip salt synthesis as well as its characterization are provided in the experimental section and ESI (Fig. S3).†

**Fig. 2:**
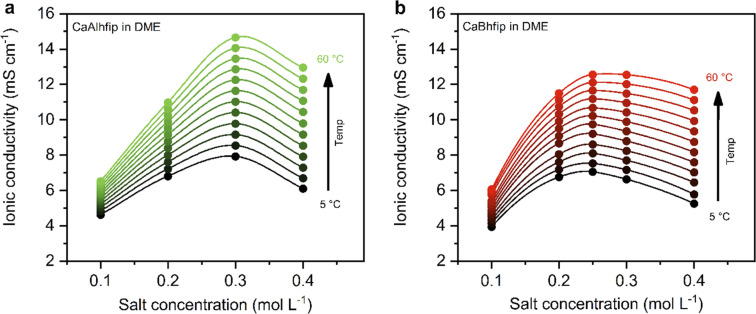
Ionic conductivity of (a) CaAlhfip and (b) CaBhfip electrolytes in DME at different temperatures, as a function of salt concentration. Lines added as visual aids, while the marked points indicate experimental data points.

The ionic conductivity, as a key parameter quantifying the ion mobility in an electrolyte, was measured for CaAlhfip and CaBhfip electrolytes in DME as a function of salt concentration and temperature (Fig. 2). In agreement with previous single-point measurements, the ionic conductivity of CaBhfip electrolytes lies in the range of 8–9 mS cm^−1^ at room temperature (0.25 M),^15^ while the Alhfip-based electrolyte displays higher ionic conductivities, in the range of 9–10 mS cm^−1^. The enhanced ionic conductivity of the alkoxyaluminate salt can be attributed to a lower tendency of this anion to form contact ion-pairs and aggregates as well as the smaller anion size, as compared to the alkoxyborate.^14,24^ As is typically observed, the ionic conductivity of both electrolytes initially increases with the salt concentration, as more charge carriers are added to the solution. At high concentrations, however, the viscosity of the electrolyte significantly rises (Fig. S4†) and the cation–anion interactions become more prominent, both phenomena hampering the ion mobility, causing a decrease in ionic conductivity. The balance between these two opposite trends results in a maximum in ionic conductivity at concentrations ∼0.25 M for CaBhfip and ∼0.3 M for CaAlhfip, similar to the case of the Mg analogues.^23^ By increasing the temperature, the viscosity of the electrolyte drops, an effect that is more evident at high concentrations (Fig. S4†). In all cases, the ionic conductivity of CaAlhfip electrolytes is higher than the one of CaBhfip electrolytes, even when the viscosity is similar. This effect is more clearly observed when constructing a Walden plot (log *∧*_eq_*vs.* log *η*^−1^), as depicted in Fig. S5.†

Based on the ionic conductivity results, 0.3 M concentration was chosen to perform electrochemical tests of both salts in DME solvent and evaluate their feasibility for calcium metal plating and stripping.

**Fig. 3:**
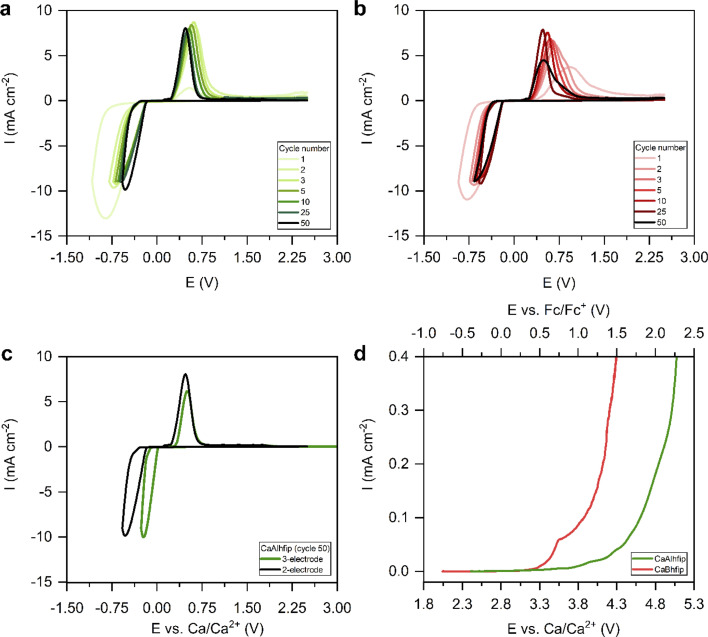
Selected CV cycles of Ca plating/stripping with a 2-electrode cell setup (Ca CE, SS WE) at 25 mV s^−1^ in (a) 0.3 M CaAlhfip/DME and (b) 0.3 M CaBhfip/DME; (c) cycle 50 of Ca plating/stripping in 0.3 M CaAlhfip/DME electrolyte with a 2- (black) and 3-electrode (green) cell setup at 25 mV s^−1^; (d) LSV in 0.3 M CaAlhfip/ACN (green) and 0.3 M CaBhfip/ACN (red) with a 3-electrode cell setup (Ca CE, SS WE, Ag RE) at 0.01 mV s^−1^. Potential of the silver reference electrode is calibrated with respect to the ferrocene redox couple.

Electrochemical performance of the novel CaAlhfip electrolyte was first evaluated with cyclic voltammetry (CV) (Fig. 3a). Instead of traditional CV settings, where one determines the cycling potential window with a lower and upper cut-off potential, we adapted the parameters by limiting the plating current density (8.85 mA cm^−2^) and using an upper cut-off voltage limit of 2.5 V *vs.* the Ca metal counter electrode. The current density limitation offers more comparable conditions when testing different electrolytes since the plating part of the cycle occurs at comparable current densities and also the amount of the transferred charge is more comparable. The measurement was first performed in a 2-electrode cell with the Ca metal as the counter electrode and stainless steel (SS) as the working electrode (WE). Selection of the working electrode was already shown to play an important role influencing the Ca plating/stripping efficiency.^26^ As noble metal current collectors can potentially catalyze electrolyte decomposition reactions through proton abstraction^27^ or form alloys with the Ca metal,^28^ SS as the most inert current collector was used in electrochemical measurements within this work. In the first cycle, the CaAlhfip electrolyte displayed a large plating overpotential and poor Coulombic efficiency (8%), which is attributed to the Ca anode activation process. In the second cycle, a significant improvement in the cell performance was observed with a Coulombic efficiency for Ca stripping and plating of 62% and reduction of cell overpotential. In latter cycles, electrochemical performance increased further and reached its maximum value in the fifth cycle (70%), followed by stable cycling with only slight fading. Coulombic efficiency of Ca plating/stripping after 50 cycles remained above 65% (Fig. S6†). The cycling performance of 0.3 M CaBhfip in DME is shown in Fig. 3b. One should note that the CaBhfip electrolyte performance reported in this work differs from previously published studies, which can be explained by the difference in the electrochemical cell setup and by the selection of different working electrodes.^26^ The CV plot of the CaBhfip/DME electrolyte exhibits an initial activation period that lasts for 6 cycles, after which the Coulombic efficiency for Ca plating and stripping stabilizes around 65%. After 25 cycles, performance starts to decrease due to the gradual passivation (Fig. S6†). Although change of the anion from boron-based to aluminum one did not affect the Coulombic efficiency and overpotential as significantly as in the Mg system,^24^ comparison of CaBhfip and CaAlhfip reveals improved performance of the latter, especially considering its long-term cycling stability.

Additionally, CV cycling experiments in both electrolytes were performed in the 3-electrode cell setup with the Ca counter, SS working, and Ag reference electrodes (Fig. S7†). Ag wire was calibrated with the ferrocene couple, which displayed a stable reversible potential at 0.16 V *vs.* Ag wire (Fig. S8†). The CaAlhfip electrolyte again first displays lower Coulombic efficiency, which after the activation process in initial cycles increases and stabilizes around 60%. This is lower compared to the 2-electrode cell and could be connected with a larger amount of electrolyte used in a 3-electrode cell setup and with it connected enhanced passivation of Ca metal deposits due to the increased amount of impurities. Comparing the performance of both electrolytes, similar trends are observed as in the 2-electrode setup. The CaAlhfip electrolyte outperforms the CaBhfip electrolyte displaying higher current densities and 0.1 V lower deposition overpotential after 50 cycles. The main difference observed comparing 2- and 3-electrode cell measurements in each electrolyte is the Ca plating and stripping voltage hysteresis. The comparison in the CaAlhfip electrolyte is given in Fig. 3c. The significantly higher voltage hysteresis for Ca plating/stripping recorded in 2-electrode cells (1 V), when compared with the 3-electrode configuration (0.6 V) points to an important contribution of the Ca counter electrode, which is especially pronounced in the cathodic part of sweep, and highlights the importance of the use of appropriate electrochemical setups.

The oxidative stabilities of CaAlhfip and CaBhfip salts were examined with linear sweep voltammetry (LSV) on the SS working electrode at a slow scan rate of 0.01 mV s^−1^ (Fig. 3d). Since glyme-type of solvents have very limited oxidative stability,^29^ determination of oxidative stability was performed in acetonitrile (ACN), which exhibits significantly higher oxidative stability, above 5 V *vs.* Li^+^/Li (corresponding to approximately 2 V *vs.* Fc/Fc^+^).^30^ The stability of the CaAlhfip/ACN electrolyte was determined at 0.8 V *vs.* Fc/Fc^+^, while the stability of CaBhfip/ACN was lower and displayed an onset of decomposition at 0.3 V *vs.* Fc/Fc^+^. Accordingly, CaAlhfip shows around 0.5 V higher oxidative stability compared to the CaBhfip analogue. Experimental results are in good agreement with the calculations of HOMO energy levels for Bhfip^−^ and Alhfip^−^ anions (−7.84 and −8.23 eV *vs.* vacuum), suggesting improved oxidative stability of the Alhfip^−^ anion.^23^

**Fig. 4:**
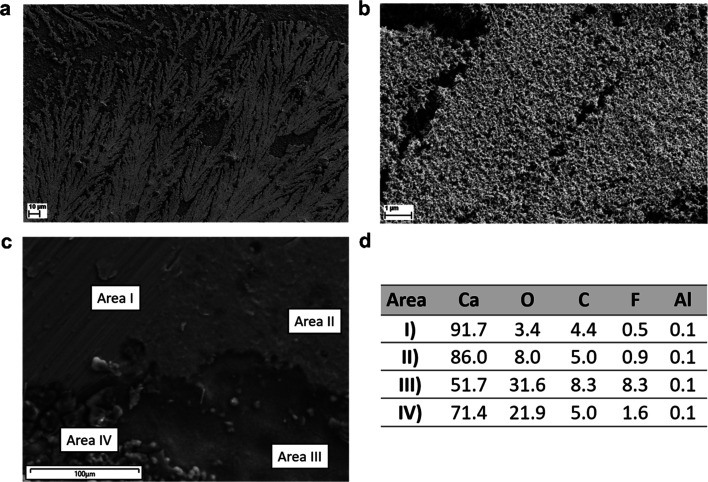
(a and b) SEM image of Ca deposits from the CaAlhfip/DME electrolyte on the carbon-coated Al foil at different magnifications revealing a dendrite-like morphology. (c) SEM image of the Ca metal electrode from the Ca‖Ca symmetric cell in the CaAlhfip/DME electrolyte with marked areas with different morphologies (I–IV). (d) Corresponding EDX measurements.

Ca metal deposits from the CaAlhfip/DME electrolyte were plated on the carbon-coated Al foil used as a working electrode and investigated using SEM and EDX analyses. Initial attempts to plate Ca metal on the SS working electrode resulted in poor adhesion of Ca metal deposits, which were instantly washed away from the electrode and could not be directly transferred to the SEM holder. A possible cause for lowered Ca plating/stripping efficiency is due to poor mechanical adhesion of Ca deposits on the electrode. Morphology investigation of Ca deposits reveals that Ca metal did not plate uniformly, but mostly in the form of dendrites (Fig. 4a and b). According to EDX analysis (Table S1†), Ca deposits consisted of only 50 wt% of Ca metal (average of 5 measured areas), while the content of oxygen was unexpectedly high, almost 30 wt%. Ca metal deposits were handled under an Ar atmosphere; therefore, the source of oxygen can be only solvent molecules that decompose on Ca metal and form an oxygen-rich surface passivation layer. Effect of solvent washing after cell disassembly was evaluated using EDX analysis on a piece of fresh Ca metal dipped into DME solvent. The Ca metal content remained close to 90 wt% (Fig. S9a†), which confirmed solvent decomposition on the surface of Ca metal, but at a significantly lower degree than in the case of Ca metal deposits. To eliminate the possibility of oxygen contamination during sample transfer, we also analyzed a fresh piece of Ca metal (Fig. S9b†), which displayed only 2.7 wt% of oxygen. The large surface of Ca deposits and highly reactive surface of the electroplated Ca could lead to more intense electrolyte decomposition on the surface of Ca deposits. A similar observation was also obtained in the case of the CaBhfip electrolyte, where the Ca content was around 50 wt%, and the oxygen content was above 30 wt% (Table S2†). This is in agreement with results reported from initial studies of the CaBhfip electrolyte, although the high oxygen amounts were previously attributed to potential air exposure during sample transfer.^15^ Another interesting observation is relatively low amounts of F and Al pointing to the relatively good stability of the salt anion, which is again in contrast to previous reports, where CaF_2_ was identified as one of the main side products.^15,16^ In this work, the determined F content in deposits from both electrolytes is below 10 wt% (9.4 and 6.6 wt%), whereas CaAlhfip displays a higher amount of F. Some deviations could derive from incomplete electrode washing before measurements. The relatively low F content in both electrolytes is attributed to a low degree of ion pairs, which is in agreement with determined physicochemical properties. As reported by Jankowski *et al.*, a low degree of ion pairs prevents anion defluorination.^31^ Therefore, decomposition of coordinated solvent molecules from the cation solvation shell is identified as the main contribution to side reactions at the Ca metal/electrolyte interface leading to a solvent dominated passive layer.

To further investigate Ca plating/stripping, we cycled symmetrical Ca‖Ca cells and studied the surface of Ca metal electrodes after CV cycling with limited plating current density (8.85 mA cm^−2^) and an upper cut-off voltage of 1 V at 25 mV s^−1^ for 10 cycles (Fig. S10†). The SEM image of the cycled Ca electrode is shown in Fig. 4c. Several distinct morphologies can be easily observed on the surface of the Ca metal electrode. Certain areas (Area I) exhibit smooth metal morphology that can be traced to the surface of the pristine Ca metal electrode and do not show any evidence of undergoing electrochemical reaction displaying a high Ca content (92%) (Fig. 4d), similar to the Ca metal that was only dipped into DME solvent. Aside from this seemingly non-active area, there are also areas (Area II), covered by a relatively thick film, where the Ca content is somewhat lowered (86%), but still remains relatively high. Two types of surfaces can be observed over the area with the increased surface roughness caused by electrochemical plating and stripping of Ca metal. On one side, we have pits covered by the surface film (Area III) and pieces of redeposited Ca metal (Area IV). Both display a decreased Ca content, which is in pits around 71%, while pieces of redeposited Ca metal display 52% of Ca similar to Ca metal deposits on the carbon coated Al electrode (Fig. 4a and b). This confirms that Ca metal plating/stripping is not uniform over the whole Ca electrode and supports the conclusion that electrochemically deposited Ca metal is more prone to side reactions with the electrolyte than pristine Ca metal, which results in a lowered Ca content of deposits.

**Fig. 5:**
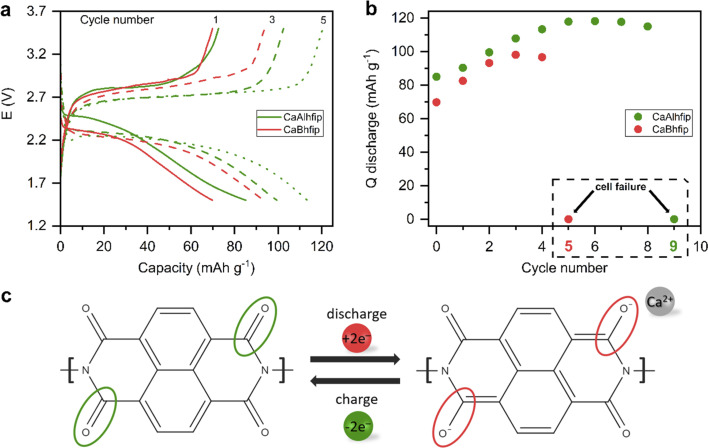
(a) Selected discharge/charge profiles of the Ca metal-NP battery in CaAlhfip/DME (green) and CaBhfip/DME (red) at 50 mA h g^−1^ and the voltage window from 1.5 to 3.5 V. (b) Discharge capacities with marked cycles of cell failure. (c) Schematic of the discharge/charge cycle.

The CaAlhfip electrolyte was evaluated in a full Ca metal anode battery cell with an organic cathode (Fig. 5). The Ca^2+^ ion has a bivalent nature and a relatively small ion radius, resulting in high charge density, which is why Ca^2+^ ion insertion into inorganic hosts is rather difficult and only the VS_4_ inorganic cathode had so far demonstrated practical electrochemical reversibility.^32,33^ On the other hand, organic materials based on the polymer group have already exhibited good electrochemical performance and long-term stability with a variety of multivalent cations.^25,34^ Among different organic polymers polyimides are especially interesting due to their high thermal stability and good mechanical properties. Full cell performance with the CaAlhfip electrolyte was evaluated in combination with the nanostructured naphthalene-hydrazine diimide polymer-based cathodes (NP) with a theoretical capacity of 203 mA h g^−1^. The polymer was nanostructured using multiwalled carbon nanotubes to improve the electrochemical accessibility of electroactive groups (Fig. S11†).^19^ The CaAlhfip/DME electrolyte in the Ca-NP battery configuration exhibits low capacity in the initial cycle (85 mA h g^−1^), which is gradually increasing, reaching 118 mA h g^−1^ in cycle 5, corresponding to more than 50% utilization of the active material. Afterwards, capacity exhibits slow and gradual fade, but in the 9th cycle, a sudden drop to only 0.05 mA h g^−1^ is observed (Fig. S12†). Inferior performance was observed in the CaBhfip electrolyte, where a sudden capacity drop occurred already in the 5th cycle (Fig. S13†). The reason for the sudden capacity drop is attributed to the increase of overpotential on the Ca metal anode electrode during the Ca stripping process, which was already observed before.^19^ Due to the short cycle life of cells in the CaBhfip electrolyte comparison with the CaAlhfip electrolyte is limited to initial cycles only (1–4). Comparing both electrolytes, CaAlhfip reaches higher capacities. The Ca metal–organic cell with the CaAlhfip exhibits a significantly lower overpotential as with CaBhfip. Another advantage of the CaAlhfip electrolyte is increase of cycle life, since it enables twice as many cycles as the boron-based analogue before cell failure. However, it is important to note that the polyimide organic cathode offers good cyclability in both electrolytes. The limited cycle life in the Ca metal cell setup can be ascribed to the limiting performance of the Ca metal anode. The latter originates from the instability of the metal/electrolyte interface leading to gradual build-up of the cell overpotential.^19^ Improved cyclability could thus be achieved in the future by designing a better Ca metal/electrolyte interface^35^ or use of an alternative anode material, which comes at a relatively high cost of losing the high capacity of the Ca metal anode.^36^

## Conclusions

In the present work, we have developed a synthesis approach towards a new calcium aluminate salt. The CaAlhfip electrolyte displays a clear advantage in terms of ionic conductivity, Ca plating/stripping stability and efficiency, and full cell performance of Ca metal–organic cells over the analogous CaBhfip electrolyte. CaAlhfip salt exhibits a high-oxidative stability of 0.8 V *vs.* Fc (0.5 V higher than CaBhfip), which makes it interesting for the development of the next-generation of high-voltage Ca electrolytes for high-voltage Ca hosts, a prerequisite for realization of high-energy density Ca battery ambition. At the same time, we show that both CaAlhfip and CaBhfip electrolytes suffer from limited stability at the surface of Ca metal, causing gradual passivation of the Ca metal anode and eventual cell failure. Ca metal passivation seems to be dominated by the decomposition of DME solvent, which identifies “an elephant in the room”, thermodynamic instability of glyme solvent in the solvation shell at the surface of Ca metal. This sets a clear challenge for the next generation of Ca electrolytes, which should prevent solvent decomposition on the surface of Ca metal and its passivation. This could be achieved by use of different, reductively more stable solvents, or engineering of the Ca solvation shell to remove DME from the first solvation shell. The second possible approach would be formation of a stable, yet Ca^2+^ cation conductive passive layer on the surface of Ca metal. Demonstration of Ca salt based on aluminate anions significantly opens the future exploration space from current borate anion-based compounds. This will greatly benefit both the synthesis of new Ca aluminate salt and exploration of passive layers incorporating aluminate groups leading to the accelerated development of Ca batteries. Higher oxidative stability of Alhfip^−^ could make it interesting for application in other battery applications, where oxidatively less stable LiBhfip salt is already being actively considered for safe and low maintenance Li-ion batteries.^37^

## Conflicts of interest

The authors declare no conflict of interest.

## Supplementary Material

TA-011-D3TA02084C-s001
