# Supplementary material for: A novel calcium fluorinated alkoxyaluminate salt as a next step towards Ca metal anode rechargeable batteries
Source: J Mater Chem A Mater. 2023 May 23;11(27):14738–47. doi: 10.1039/d3ta02084c (PMC10335333; doi:10.1039/d3ta02084c)
Supplement: TA-011-D3TA02084C-s001 [file TA-011-D3TA02084C-s001.pdf]

Electronic Supplementary Material (ESI) for Journal of Materials Chemistry A.
This journal is © The Royal Society of Chemistry 2023

Supporting Information

A novel calcium fluorinated alkoxyaluminate salt as a next step towards Ca metal anode rechargeable batteries

Tjaša Pavčnik[1,2], Juan D. Forero-Saboya[3], Alexandre Ponrouch[3,4], Ana Robba[2], Robert Dominko[1,2,4], Jan Bitenc[1,2*]

[1]National Institute of Chemistry, Hajdrihova 19, 1000 Ljubljana, Slovenia

[2]Faculty of Chemistry and Chemical Technology, University of Ljubljana, Večna pot 113, 1000, Ljubljana, Slovenia

[3]Institut de Ciència de Materials de Barcelona, ICMAB-CSIC, Campus UAB, 08193 Bellaterra, Spain

[4]Alistore-European Research Institute, CNRS FR 3104, Hub de l'Energie, Rue Baudelocque, 80039 Amiens, France

[*]Corresponding author: jan.bitenc@ki.si

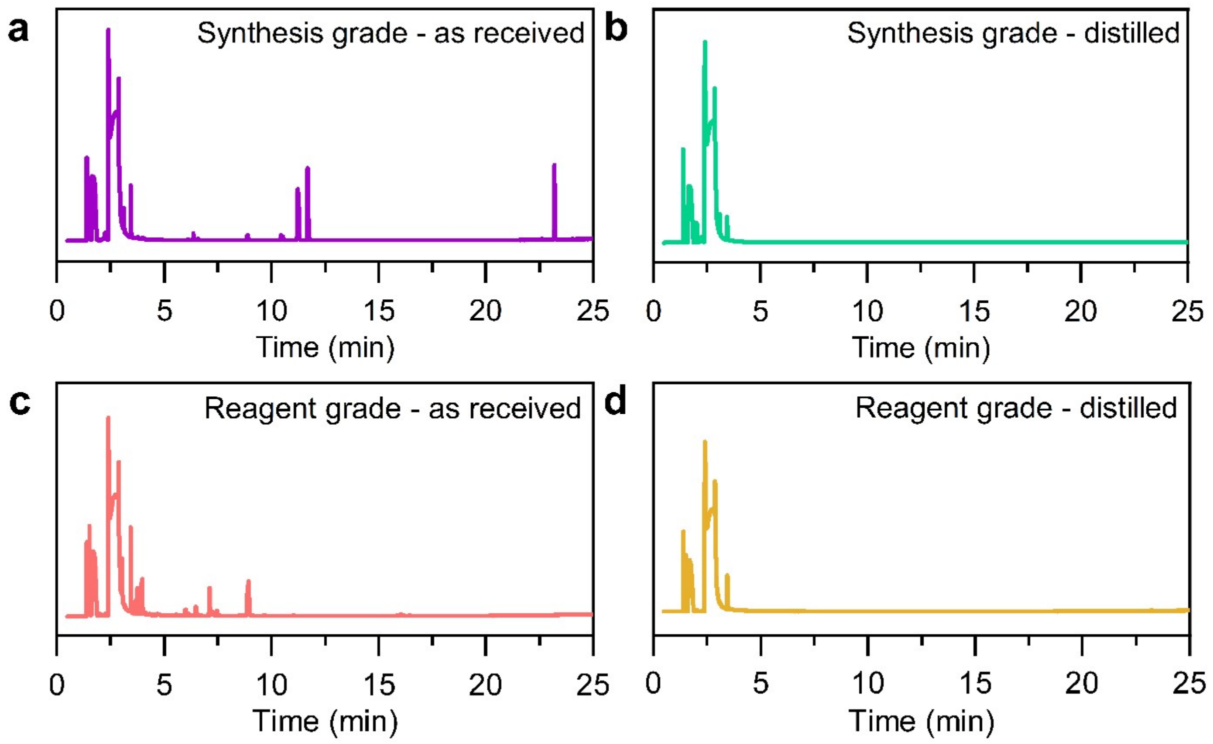

Figure S1: Chromatograms of DME solvents: synthesis grade – **a)** as received and **b)** after drying/purification procedure including fractional distillation; reagent grade – **c)** as received and **d)** after drying/purification procedure including fractional distillation.

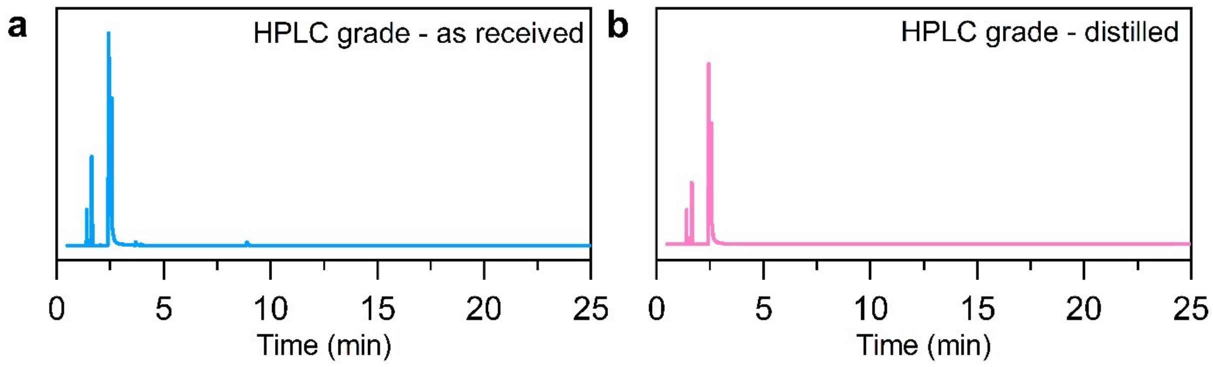

Figure S2: Chromatograms of DME solvents: chromatographic (HPLC) grade – **a)** as received and **b)** after drying/purification procedure including fractional distillation.

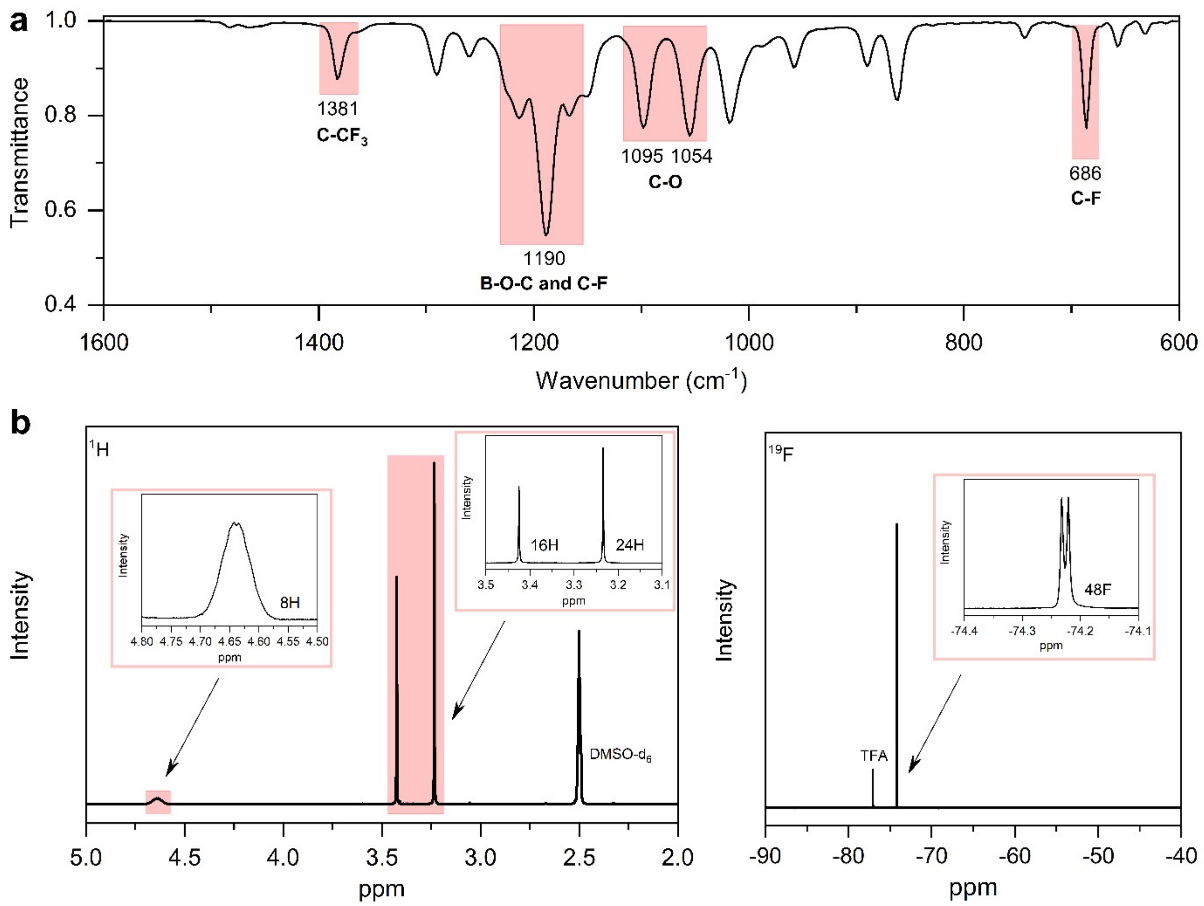

Figure S3: a) ATR-IR spectrum of CaBhfip with peak assignation. **b)** ^{1}H (right) and ^{19}F (left) NMR spectra of CaBhfip with peak assignation.

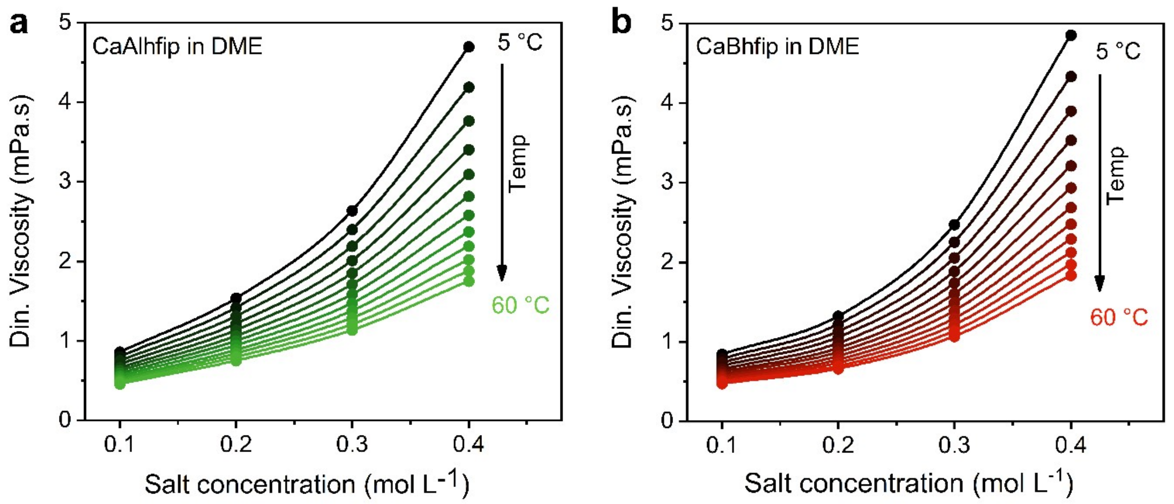

Figure S4: Viscosity of electrolytes in DME at different temperatures as function of salt concentration. **a)** CaAlhfip, **b)** CaBhfip. Lines added only as visual aid, the markers indicate experimental data points.

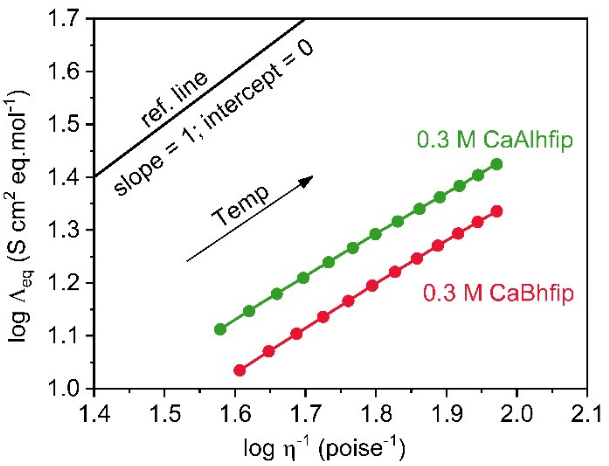

Figure S5: Walden plot of 0.3 M CaAlhfip and 0.3 M CaBhfip in DME. The plot was constructed using the viscosity and ionic conductivity of the electrolytes measured at different temperatures between 5 and 60 °C.

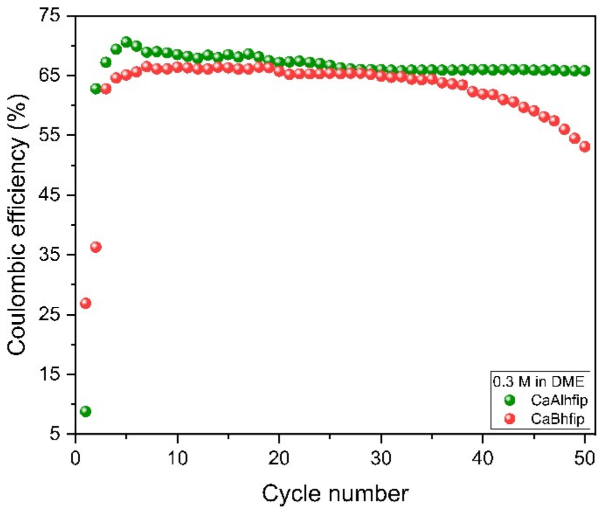

Figure S6: Coulombic efficiency for Ca plating/stripping in 0.3 M CaAlhfip and CaBhfip electrolytes in DME with a 2-electrode cell setup. The efficiencies were calculated manually from CV curves (**Figure 3**, **a** and **b**) by dividing the area under the curve for Ca stripping with the area above the curve for Ca plating.

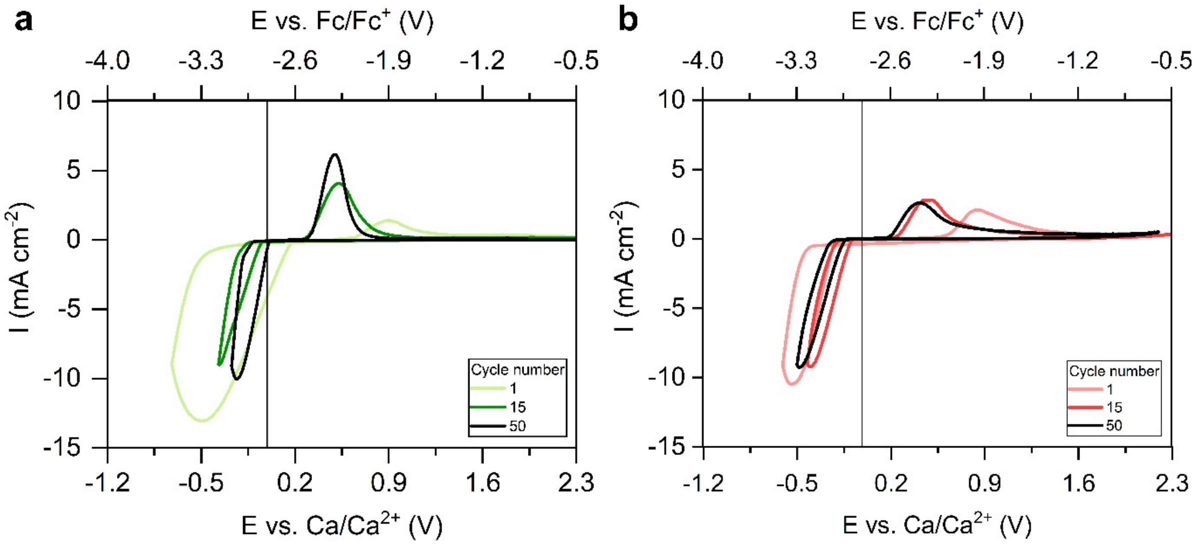

Figure S7: Selected CV cycles of Ca plating/stripping in **a)** 0.3 M CaAlhfip/DME and **b)** 0.3 M CaBhfip/DME with a 3-electrode cell setup (Ca CE, SS WE, Ag RE) at 25 mV s^{-1}. Potential of reference electrode is calibrated with respect to ferrocene redox couple.

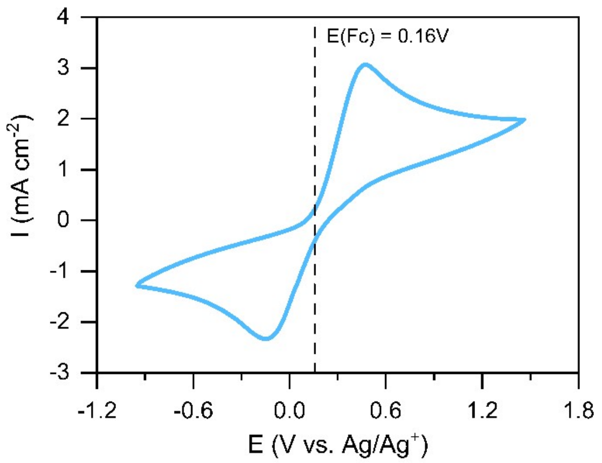

Figure S8: The redox potential of ferrocene (Fc) vs. Ag reference electrode measured in 3-electrode cell configuration using Ca as counter, SS as working and Ag as reference electrode at 25 mV s^{-1}.

Table S1: EDX analysis of deposits from 0.3 M CaAlhfip/DME electrolyte with reported average values of five measurements for each element.

No.	Ca	O	C	F	Al
**1**	58.2	17.9	6.7	13.7	3.5
**2**	50.9	20.7	8.7	5.2	2.2
**3**	46.4	31.8	8.6	11.9	1.3
**4**	47.8	30.8	8.6	11.4	1.4
**5**	45.5	35.1	13.7	4.9	0.8
**Average**	49.8	27.3	9.3	9.4	1.8

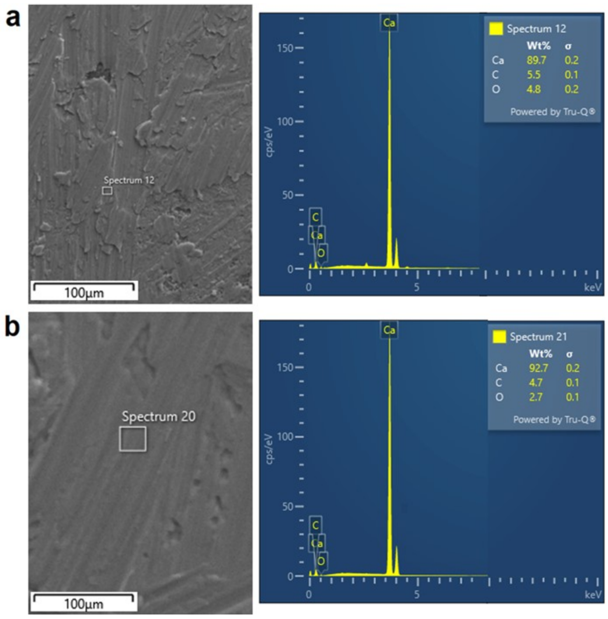

Figure S9: **a)** SEM image and corresponding EDX spectra of Ca metal dipped into DME and **b)** fresh Ca metal.

Table S2: EDX analysis of deposits from 0.3 M CaBhfip/DME electrolyte with reported average values of five measurements for each element.

No.	Ca	O	C	F
**1**	50.8	37.4	6.1	5.7
**2**	45.6	32.6	12.3	9.4
**3**	50.9	37.1	7.4	4.6
**4**	51.6	35.4	4.7	8.3
**5**	45.5	31.8	14.4	8.4
**Average**	48.9	35.3	9.2	7.3

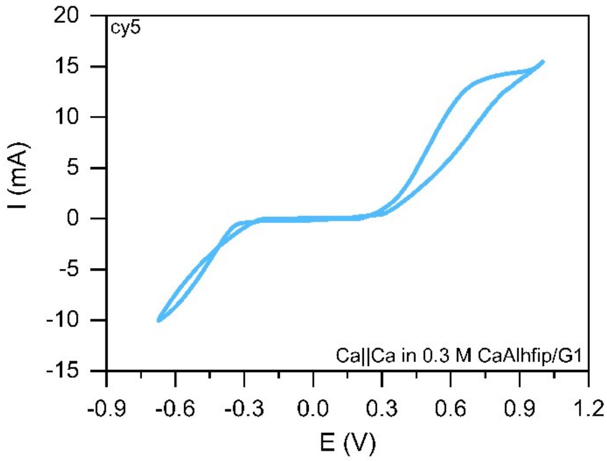

Figure S10: 5th CV cycle of Ca plating/stripping in 0.3 M CaAlhfip/DME with a 2-electrode symmetrical Ca||Ca cell (Ca CE, Ca WE) at 25 mV s^{-1}.

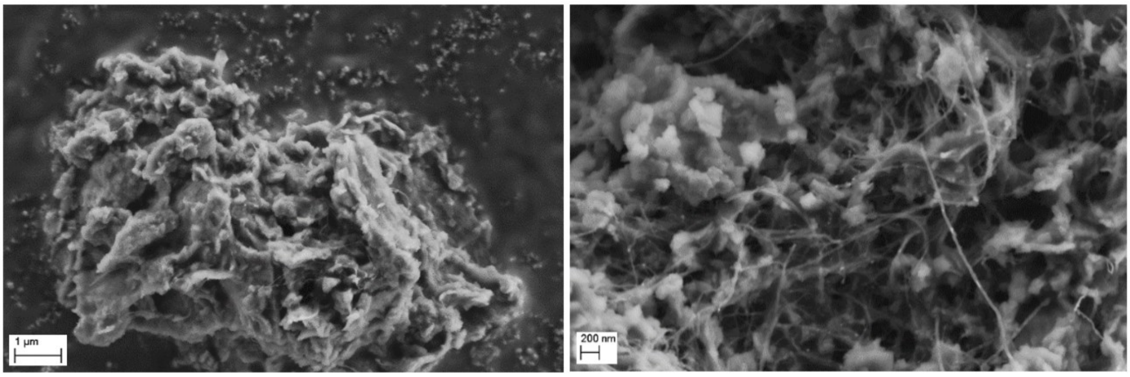

Figure S11: SEM micrographs of naphthalene-hydrazine diimide polymer nanostructurized with MWCNTs.

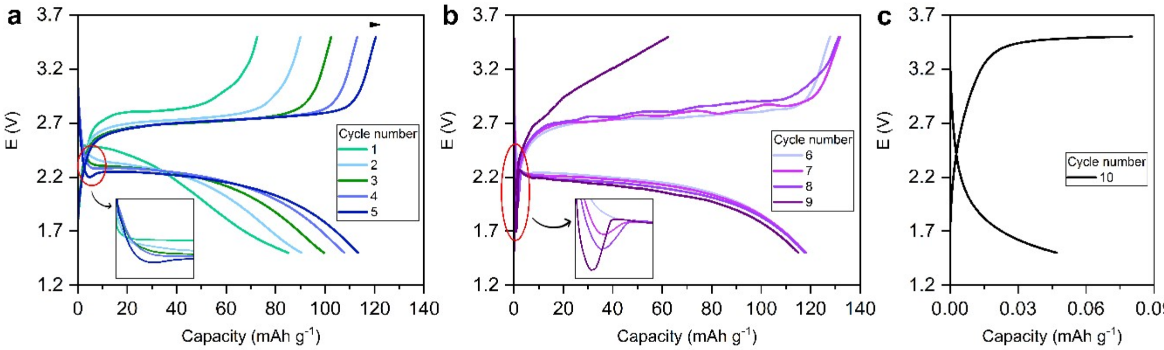

Figure S12: Discharge/charge profiles of Ca metal – NP battery in 0.3 M CaAlhfip/DME at C/4 and voltage window from 1.5 to 3.5 V: **a)** cycles 1–5; **b)** cycles 6–9; **c)** cycle 10. Inset areas represent voltage dips due to the increase of overpotential on anode.

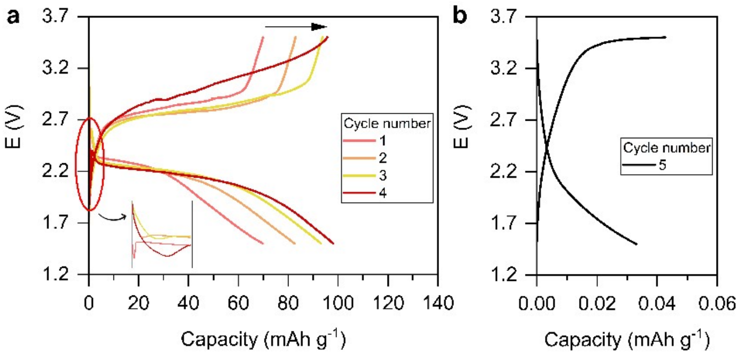

Figure S13: Discharge/charge profiles of Ca metal – NP battery in 0.3 M CaBhfip/DME at C/4 and voltage window from 1.5 to 3.5 V: **a)** cycles 1–4; **b)** cycle 5. Magnified areas represent voltage dips due to the increase of overpotential on anode.
